# Preparing for the unexpected: special considerations and complications after sugammadex administration

**DOI:** 10.1186/s12871-017-0429-9

**Published:** 2017-10-17

**Authors:** Hajime Iwasaki, J. Ross Renew, Takayuki Kunisawa, Sorin J. Brull

**Affiliations:** 1 Department of Anesthesiology and Critical Care Medicine, Asahikawa Medical University, 2-1-1-1 Midorigaoka-higashi, Asahikawa, Hokkaido 078-8510 Japan; 2 Department of Anesthesiology and Perioperative Medicine, Mayo Clinic, 4500 San Pablo Rd, Jacksonville, Florida, 32224 USA

**Keywords:** Neuromuscular blocking drugs, Neuromuscular function, Neuromuscular block, Sugammadex, Neostigmine, Pharmacologic reversal, Anticholinesterase

## Abstract

Sugammadex, a modified gamma-cyclodextrin, has changed clinical practice of neuromuscular reversal dramatically. With the introduction of this selective relaxant binding agent, rapid and reliable neuromuscular reversal from any depth of block became possible. Sugammadex can reverse neuromuscular blockade without the muscarinic side effects typically associated with the administration of acetylcholinesterase inhibitors. However, what remained unchanged is the incidence of residual neuromuscular blockade. It is known that sugammadex cannot always prevent its occurrence, if appropriate dosing is not chosen based on the level of neuromuscular paralysis prior to administration determined by objective neuromuscular monitoring. Alternatively, excessive doses of sugammadex administered in an attempt to ensure full and sustained reversal may affect the effectiveness of rocuronium in case of immediate reoperation or reintubation. In such emergent scenarios that require onset of rapid and reliable neuromuscular blockade, the summary of product characteristics (package insert) recommends using benzylisoquinolinium neuromuscular blocking agents or a depolarizing agent. However, if rapid intubation is required, succinylcholine has a significant number of side effects, and benzylisoquinolinium agents may not have the rapid onset required. Therefore, prior administration of sugammadex introduces a new set of potential problems that require new solutions. This novel reversal agent thus presents new challenges and anesthesiologists must familiarize themselves with specific issues with its use (e.g., bleeding risk, hypermagnesemia, hypothermia). This review will address sugammadex administration in such special clinical situations.

## Background

Sugammadex, a modified gamma-cyclodextrin, has made neuromuscular reversal faster [1] and safer [2] when compared to traditional acetylcholinesterase inhibitors. However, simply administering sugammadex based on clinical signs or time since the last administration of neuromuscular blocking agents (NMBAs) (without using objective neuromuscular monitoring to guide appropriate dosing) cannot ensure full and reliable recovery and patient safety. Residual neuromuscular paralysis is a remaining complication even with the use of sugammadex [3]. Appropriate dosing [4 mg/kg at post tetanic count (PTC) of 1–2; 2 mg/kg at reappearance of two twitches to train of four (TOF) stimulation; 1 mg/kg [4] at reappearance of four twitches to TOF stimulation; 0.49 mg/kg [5] at TOF ratio (TOFR) of ≥0.2; and 0.22 mg/kg [6] at TOFR of ≥0.5] is necessary; excessive doses of sugammadex may prevent residual neuromuscular paralysis, but may also result in excessive costs and pose an entirely new set of challenges when re-establishment of neuromuscular blockade is needed [7]. Moreover, aspects of the clinical use of sugammadex are controversial or may require special attention in some clinical situations. For example, hypersensitivity reaction to sugammadex is a life-threatening problem for which immediate detection and treatment are imperative. Anesthesiologists must familiarize themselves with these specific issues. This review will address several clinical scenarios to which attention should be focused following sugammadex administration.

## Review:

### Recurrence of neuromuscular blockade after reversal

Recurrence of neuromuscular blockade (also known as “re-curarization,” with historic reference to the use of curare), is defined as a decrease in the TOFR from “equal to or greater than 0.9” to “less than 0.8” in at least 3 consecutive TOF values [8, 9]. Two of 10 patients who received 1 mg/kg of sugammadex at a depth of block of 1–2 PTCs met this definition in a Phase 2 trial [8, 10]. Three patients in the same trial and one pediatric patient in a case report [11] did not completely meet the criteria for recurrent block, however, a temporary decrease in twitch response following sugammadex reversal was observed. In these reports, all patients had received less than the 4 mg/kg recommended dose of sugammadex (0.5 or 1 mg/kg) at a depth of block of 1–2 PTCs [8, 10, 11]. The mean time from sugammadex administration to recovery of TOFR >0.9 (prior to recurrence) was 6.9 (range 3.6–11.5) minutes [8]. The mean time to maximum decrease (after once recovered to TOFR >0.9) in twitch response after sugammadex administration (recurrence of paralysis) was 36.1 min (range 17–91) [8, 10, 11].

There are reports of recurrence of neuromuscular blockade after sugammadex reversal in obese patients [9, 12]. These patients likely had received insufficient doses of sugammadex (1.74 mg/kg when TOF count was 2 [12]; and 2 mg/kg when PTC was 1–5 [9]). One patient required five minutes for recovery to TOFR >0.9, and symptoms of recurrent weakness occurred 20 min after sugammadex administration [12]. Because of concerns of insufficient dose administration in the obese patient, current recommendations in the sugammadex summary of product characteristics (SmPC) are to administer sugammadex on total body weight (TBW) basis (not ideal body weight, IBW). This dosing, however, remains controversial [13–18].

Recurrence of neuromuscular blockade after sugammadex administration can be explained by two processes: the first is redistribution of rocuronium from peripheral and effect-site compartments (neuromuscular junction) to central (intravascular) compartment; the second is the lack of sufficient, unbound (free) sugammadex molecules in the plasma. After sugammadex administration, rocuronium molecules rapidly move from neuromuscular junction to plasma due to the concentration gradient between these two locations [19]. However, if the dose of sugammadex was insufficient to encapsulate all rocuronium molecules in the plasma, unbound rocuronium will move back into neuromuscular junction along concentration gradients, resulting in recurrent paralysis. The mean recovery time to TOFR >0.9 after the recommended dose (4 mg/kg) of sugammadex from a PTC = 1–2 is reported to be 2.8 min [20]. It seems that there is a risk of recurrent weakness after sugammadex administration when neuromuscular recovery requires more than 3 min [8, 10]. To avoid the risk of such recurrence, an appropriate dose of sugammadex based on the particular depth of neuromuscular blockade [4, 6] should always be used, and complete recovery of neuromuscular function should be confirmed with neuromuscular monitoring (ideally, objective neuromuscular monitoring).

### Residual paralysis after sugammadex administration

Residual paralysis caused by the use of muscle relaxants during surgery, or incomplete neuromuscular reversal, is associated with postoperative complications such as hypoxemia and upper airway obstruction [21–23]. It also has a strong association with pulmonary complications and morbidity in postoperative patients [24, 25]. Neuromuscular recovery to TOFR ≥0.9 is important because below this level of recovery pharyngeal dysfunction remains, increasing the risk of pulmonary aspiration when TOFR values are 0.7–0.8 measured at the adductor pollicis muscle [26, 27]. Therefore, current recommendations include confirmation of sufficient neuromuscular recovery to TOFR ≥0.9 by electromyography, and acceleromyographic recovery of TOFR ≥1.0 [28–30]. The incidence of residual paralysis is reported to be 20–60% of patients upon arrival in the post anesthesia care unit (PACU) when non-depolarizing muscle relaxants were used during the surgery [30–33]. Brueckmann et al. reported that all patients receiving sugammadex for neuromuscular blockade reversal had TOFR ≥0.9 at PACU admission, while 43% of patients treated with neostigmine / glycopyrrolate had a TOFR <0.9 at PACU arrival [34]. However, Unterbuchner [35] and Todd [36] have reported that high incidence of residual paralysis after antagonism with neostigmine was likely due to inappropriate intraoperative neuromuscular monitoring in this study. Kotake et al. reported that the use of sugammadex did not eliminate the risk of residual paralysis (up to 9.4% of the patients showed TOFR <0.9 after tracheal extubation) when the decision to extubate the trachea was not based on objective neuromuscular monitoring [3]. It is uncontroversial that neuromuscular reversal with sugammadex decreases the incidence of residual paralysis compared to acetylcholinesterase inhibitors [2, 32]. Moreover, unrestricted use of sugammadex might decrease postoperative pulmonary complications [37, 38]. However, it is imperative that a sufficient number of molecules of sugammadex be administered to bind all of the free rocuronium molecules that diffuse from the neuromuscular junction back into the plasma. The only way to estimate the quantity of these remaining unbound rocuronium molecules is to monitor neuromuscular function and determine more precisely the most appropriate dose of sugammadex necessary for complete antagonism. Therefore, quantitative intraoperative neuromuscular monitoring is strongly recommended to prevent postoperative residual paralysis [3, 39–41]. Without such objective monitoring, even the unrestricted use of sugammadex cannot completely eliminate the risk of residual paralysis [42]. Consequently, the purpose of neuromuscular monitoring is dual: on the one hand, the most effective dose should be administered in order to exclude residual paralysis. On the other hand, the lowest effective dose should be administered to ensure that excessive sugammadex does not later interfere with the potential need to re-establish neuromuscular blockade emergently. This topic will be discussed in the next section.

### Planned re-establishment of neuromuscular blockade after sugammadex administration

There are three options to re-establish neuromuscular blockade after sugammadex administration: re-administration of rocuronium; use of benzylisoquinolinium NMBAs, and use of succinylcholine [43].

**Fig. 1:**
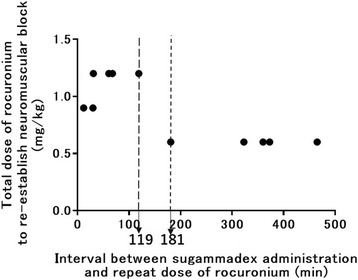
The relationship between the total dose of rocuronium (mg/kg) needed to re-establish neuromuscular blockade and time interval (minutes) between sugammadex administration and re-administration of rocuronium. It was adapted from Iwasaki et al. [7]. In this study, 0.6 mg/kg re-established neuromuscular blockade within 3 min when it was administered >3 h after sugammadex reversal. Larger doses (0.9 or 1.2 mg/kg) were necessary to re-establish neuromuscular blockade when sugammadex was administered less than 2 h previously

Sugammadex itself is not metabolized, and most of it will be excreted in urine unchanged. The rate of clearance of sugammadex is similar to the glomerular filtration rate, and its elimination half-life is approximately 100 min [44, 45]. Therefore, when rocuronium is administered soon after neuromuscular reversal with sugammadex, unbound (free) sugammadex molecules left in the circulation have the potential to bind to the administered rocuronium. In such a scenario, re-onset time of rocuronium might be prolonged and duration of action might be shortened [46]. However, re-administration of rocuronium following sugammadex reversal has the advantage that it can still be antagonized by another dose of sugammadex when recovery of function is needed. Cammu et al. reported that re-onset of neuromuscular blockade took longer (mean 3.09 min, range 1.92–4.72 min), especially when rocuronium was administered <25 min after sugammadex reversal, even after a larger than recommended dose (1.2 mg/kg) [46]. In a clinical study, the recommended dose (0.6 mg/kg) of rocuronium re-established neuromuscular blockade within 3 min when it was administered >3 h after sugammadex reversal (Fig. 1) [7]. Since re-onset time of rocuronium after sugammadex administration is unpredictable, 1.2 mg/kg rocuronium or more should be used when reliable and rapid induction of neuromuscular blockade is needed. In clinical settings where rapid induction of neuromuscular blockade is not absolutely necessary and if more than 3 h have passed after the initial sugammadex administration, utilizing rocuronium at a dose of 0.6 mg/kg becomes an additional option to re-establish neuromuscular blockade [7].

According to the sugammadex SmPC, it is recommended that benzylisoquinolinium NMBAs be used if neuromuscular blockade is required within 24 h of sugammadex administration because of pharmacological and physiological reasoning, since sugammadex has no effects on neuromuscular blockade induced by these NMBAs [47, 48]. However, benzylisoquinolinium NMBAs such as cisatracurium are not suitable for rapid sequence intubation because of relatively prolonged onset time [49, 50]. Moreover, the use of benzylisoquinolinium NMBAs to re-establish neuromuscular blockade preclude the use of sugammadex for reversal of a second neuromuscular blockade.

Since sugammadex has no affinity for encapsulating succinylcholine, unbound sugammadex after reversal should not affect the re-onset of neuromuscular blockade by succinylcholine. Asakura et al. reported that 1 mg/kg succinylcholine re-established neuromuscular blockade within 85 s, 3 h after sugammadex administration [51]. However, residual unbound non-depolarizing muscle relaxants may cause resistance to the effects of succinylcholine [52]. Therefore, the use of a larger dose of rocuronium (or benzylisoquinolinium NMBAs) may be a better option in situations in which residual paralysis is suspected (e.g. sugammadex reversal without neuromuscular monitoring during the initial surgery). Furthermore, non-depolarizing muscle relaxants will be necessary if maintenance of neuromuscular blockade is needed during reoperation.

**Fig. 2:**
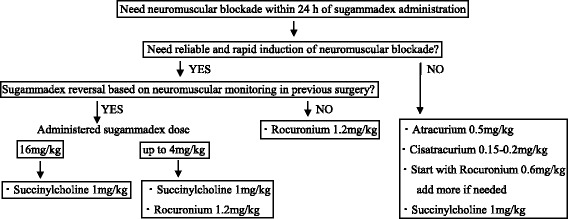
The recommended algorithm to re-establish neuromuscular blockade after sugammadex administration [46, 51, 53]

The first important point in re-establishing neuromuscular blockade after sugammadex administration is to decide whether rapid sequence induction and intubation is necessary or not. The recommended algorithm to re-establish neuromuscular blockade after sugammadex administration is shown in Fig. 2 [46, 51, 53].

### Hypersensitivity/ allergy associated with sugammadex administration

The incidence of anaphylaxis has been increasing over the past decade, with a rate between 1/10,000 and 1/20,000 anesthetics [54]. There are several reports of sugammadex-induced hypersensitivity or anaphylaxis [55–61]. There are reports that not only sugammadex itself but rocuronium-sugammadex complex could be the trigger of anaphylactic reactions [59, 61]. The most frequent symptoms of sugammadex-induced anaphylaxis are rash, hypotension and tachycardia. There is a report of transient third-degree atrio-ventricular block that was attributed to an allergic reaction to sugammadex [62]. In a recent review, most anaphylactic reactions were triggered within 4 min after sugammadex administration [63]. Most of the patients experiencing hypersensitivity reactions had no previous exposure to sugammadex. It seems likely that prior exposure to cyclodextrins, which are present in various foods, may explain the cross-reaction with sugammadex. This is consistent with the 350% increase in the rate of hospital admissions due to food-induced anaphylaxis that was reported in Australia [64]. Sadleir et al. described a protocol to test for sugammadex anaphylaxis [65]. It has to be considered that not only sugammadex itself but also the sugammadex-rocuronium complex could be the allergen of the anaphylaxis. Treatment of sugammadex-induced anaphylaxis is not different from that of other allergens [66]. The use of vasopressors and fluid administration are effective in most cases, and no fatalities related to sugammadex-induced anaphylaxis have been reported to date. Reduction in the number of free sugammadex molecules in the plasma by appropriate dosing may alleviate the severity of anaphylaxis [60].

### Anticoagulant effect after sugammadex administration

Because a prior in vitro study demonstrated sugammadex concentration-dependent increases in prothrombin time (PT) and activated partial thromboplastin time (APTT) [67], several clinical studies have been performed. De Kam et al. found no clinically relevant reduction in platelet aggregation after 4 mg/kg sugammadex following oral aspirin 75 mg in healthy subjects [68]. The same authors also found no clinically meaningful effects to either anti-Xa activity or APTT after 4 or 16 mg/kg sugammadex following pretreatment with enoxaparin or unfractionated heparin [69]. These findings suggest that increased APTT and PT following 4 or 16 mg/kg sugammadex are transient and are unlikely to be clinically relevant. Recently, Dirkmann et al. reported that sugammadex affects various coagulation assays by binding phospholipids contained in such assays [70]. They concluded that the prolongation of coagulation parameters caused by sugammadex may be just an in vitro artifact [70].

The effects of sugammadex and neostigmine on postoperative bleeding risk were compared in several publications. In a randomized double-blind study of patients undergoing orthopedic surgery, Rahe et al. compared sugammadex 4 mg/kg with usual care (neostigmine or spontaneous recovery) on postoperative bleeding events within 24 h postoperatively [71]. Despite transient (within 1 h) increases in APTT and PT in the sugammadex group, there was no increased risk of bleeding versus usual care. In another study of patients undergoing septoplasty, the authors evaluated the total amount of blood absorbed by the nasal dressings in the first 3 postoperative hours [72]. In this study, no statistically significant difference was found in PT and APTT values between the patients who received 2 mg/kg sugammadex and those who received neostigmine. However, the amount of postoperative blood loss was statistically significantly higher in the sugammadex group (4.13 mL) compared to the neostigmine group (2.48 mL). It is unlikely, however, that 2–3 mL difference in blood loss is clinically significant, even in septal surgery.

Although sugammadex increases some laboratory coagulation parameters transiently, the use of sugammadex has not resulted in clinically significant postoperative bleeding.

### Sugammadex reversal in special situations

#### Hypermagnesemia and hypothermia

*Magnesium* administration is used in a variety of perioperative settings including the treatment of torsades de pointes [73], as a tocolytic in the parturient [74]*,* as an anticonvulsant in women with preeclampsia and eclampsia [75], and for facilitating endotracheal intubation by accelerating the onset of NMBA [76, 77]. In addition to decreasing the release of acetylcholine by inhibiting voltage-dependent calcium channels, high plasma levels of this cation also diminish the depolarizing action of acetylcholine at the motor end-plate [78]. Magnesium has also been shown to delay the reversal of vecuronium-induced neuromuscular blockade by neostigmine and may result in re-occurrence of neuromuscular blockade [79].

With the significant impact of magnesium on neuromuscular blockade and subsequent reversal with acetylcholinesterase inhibition, its effect on reversal with sugammadex has been questioned. Animal models demonstrate mixed results when investigating whether raising plasma magnesium levels impacts the dose of sugammadex needed to reverse neuromuscular blockade [80, 81]. Aside from one observational study that suggested patients may need sugammadex doses exceeding 14 mg/kg in the presence of a magnesium infusion for the treatment of HELLP syndrome [82], the remaining literature suggests that reversal with standard doses of sugammadex is not prolonged in patients receiving clinically-relevant doses of intravenous magnesium. While utilizing magnesium infusions in an effort to blunt the cardiac response to airway management, Carron et al. described using a standard dose of sugammadex to reverse moderate neuromuscular blockade in a morbidly obese patient. Complete reversal was noted within 60 s of administration [83]. Two randomized controlled trials have also demonstrated no prolongation in the recovery time from moderate and deep levels of neuromuscular blockade using standard doses of sugammadex in patients receiving magnesium boluses. Filho et al. randomly assigned 73 patients to receive either magnesium sulphate (40 mg/kg) or saline and found no difference in reversal time between the two groups following sugammadex administration [84]. In a similar study, Czarnetzki et al. randomized 32 patients to receive magnesium sulfate (60 mg/kg) or placebo. The average time for reversal of moderate neuromuscular blockade was again not significantly different between the two groups [85]. While the available clinical trials are discordant with animal models and do not suggest magnesium delays neuromuscular blockade reversal by sugammadex [86], close monitoring (preferably, using objective means) and cautious clinical judgment are still prudent as clinicians gain experience with this reversal agent.

In addition to hypermagnesemia, hypothermia is another clinical scenario implicated in prolonging recovery time from neuromuscular blockade [87]. One randomized controlled trial exists that investigated whether such interactions apply to recovery times when sugammadex is administered for the reversal of steroidal neuromuscular blocking agents. In this trial, Lee and colleagues randomized 60 patients to mild hypothermia or normothermia. While complete reversal of deep neuromuscular blockade with sugammadex was achieved in both groups, the duration of recovery time was significantly prolonged in the hypothermia group versus the normothermic group (171.1 ± 62.1 s vs. 124.9 ± 59.2 s, respectively, *p* = 0.005) [88]. The authors speculated that this delay might be caused by the decrease in cardiac output associated with hypothermia, and the resultant decrease in drug delivery to skeletal muscle groups. Acidosis and hypercarbia are also two factors that have significant implications for managing NMBA administration and reversal, although to date there are no prospective studies investigating the effects of these metabolic derangements on the effectiveness of sugammadex.

## Conclusions

As the international use of sugammadex continues to expand, new scenarios will arise that challenge clinicians, and a thorough understanding of the properties, advantages and limitations of this drug are of paramount importance. Similarly important for good clinical care and patient safety, clinicians must remember that the reversal dose of sugammadex should always be calculated based on the degree of neuromuscular recovery obtained with neuromuscular monitoring (ideally, objective neuromuscular monitoring). By doing so, the use of sugammadex eliminates recurrence and/or residual paralysis after neuromuscular reversal. Appropriate dosing of sugammadex also broadens the range of clinical options when reliable and rapid re-induction of neuromuscular blockade is needed. Even in special situations (e.g. hypermagnesemia, hypothermia), sugammadex has an advantage as a neuromuscular reversal agent compared with acetylcholinesterase inhibitors. Although hypersensitivity to sugammadex is unpredictable, such rare events typically occur within minutes of administration and should be detected and treated successfully by the vigilant clinician.

## Abbreviations

APTT: activated partial thromboplastin time
IBW: ideal body weight
NMBA: neuromuscular blocking agent
PACU: post anesthesia care unit
PT: prothrombin time
PTC: post tetanic count
SmPC: summary of product characteristics
TBW: total body weight
TOF: train-of-four
TOFR: train-of-four ratio
